# Pro-Resolving Mediators in Regulating and Conferring Macrophage Function

**DOI:** 10.3389/fimmu.2017.01400

**Published:** 2017-11-01

**Authors:** Jesmond Dalli, Charles N. Serhan

**Affiliations:** ^1^Lipid Mediator Unit, William Harvey Research Institute, Bart’s and the London School of Medicine, Queen Mary University of London, London, United Kingdom; ^2^Center for Experimental Therapeutics and Reperfusion Injury, Harvard Institutes of Medicine, Brigham and Women’s Hospital and Harvard Medical School, Boston, MA, United States

**Keywords:** lipid mediators, omega 3, microvesicles, immunoresolvent, tissue regeneration

## Abstract

Macrophages are central in coordinating the host response to both sterile and infective insults. Clearance of apoptotic cells and cellular debris is a key biological action preformed by macrophages that paves the way to the resolution of local inflammation, repair and regeneration of damaged tissues, and re-establishment of function. The essential fatty acid-derived autacoids termed specialized pro-resolving mediators (SPM) play central roles in promoting these processes. In the present article, we will review the role of microvesicles in controlling macrophage efferocytosis and SPM production. We will also discuss the role of both apoptotic cells and microvesicles in providing substrate for transcellular biosynthesis of several SPM families during efferocyotsis. In addition, this article will discuss the biological actions of the recently uncovered macrophage-derived SPM termed maresins. These mediators are produced via 14-lipoxygenation of docosahexaenoic acid that is either enzymatically converted to mediators carrying two hydroxyl groups or to autacoids that are peptide-lipid conjugates, coined maresin conjugates in tissue regeneration. The formation of these mediators is temporally regulated during acute self-limited infectious-inflammation where they promote the uptake and clearance of apoptotic cells, regulate several aspects of the tissue repair and regeneration, and display potent anti-nociceptive actions.

## Introduction

Inflammation is mounted in response to injury and/or infection in vascularized tissues that results in edema formation and leukocyte trafficking to the injured site and/or point of bacterial invasion (1). This is a fundamental host defense process that ensures adequate and timely disposal of invading pathogens and the repair of damaged tissues, paving the way for organ/tissue regain of function. At a histological level, the resolution of inflammation is characterized by the clearance of infiltrated leukocytes from the site and regain of tissue architecture (1). For many years, it was thought that the inflammatory response is terminated when local inflammatory chemical messengers and cells were *passively* diluted at the site (dilution of chemotactic gradient), hence halting further leukocyte recruitment, resolving the exudate or battlefield of inflammation (1–3). Detailed studies of cellular trafficking at the site demonstrated that in self-resolving inflammatory exudates cellular trafficking was tightly coordinated, where tissue resident cells elaborated the inflammatory reaction when exposed to an inflammatory stimulus. This was rapidly followed by an influx of granuloctyes, primarily neutrophils, and subsequently monocytes (4). In self-contained exudates, these recruited monocytes change phenotype from an inflammatory to a tissue protective phenotype as they differentiate to macrophages. This specific macrophage subpopulation is referred to as a resolution phase macrophages (5) and is thought to play key roles in the clearance of cellular debris from the site of inflammation and may also be involved in promoting tissue repair and regeneration (6–8).

These trafficking studies also suggested that since resolution is a tightly coordinated process, it was unlikely that simple dissipation of inflammatory signals could be the underlying mechanism for such a fundamental process. Findings made using a systems approach, assessing cellular trafficking and function coupled with biochemical approaches for structure elucidation of previously unknown mediators, highlight that indeed resolution of inflammation is a biochemically active process. These studies demonstrate that within exudates the production of inflammatory mediators such as leukotriene (LT) B_4_ and prostaglandin E_2_ was temporally regulated and reached a maximum at peak leukocyte infiltration. These studies also demonstrate that the resolution phase is denoted by the formation of a novel genus of autacoids that actively counter-regulate the formation of pro-inflammatory mediators, cellular trafficking, and phenotype (2, 9, 10). Given their potent biological actions, this novel genus of mediators is termed specialized pro-resolving mediators (SPM). SPM encompass several families of structurally and chemically distinct mediators. These, include neuroprotectin D1/NPD1 (10*R*,17S-dihydroxy-4Z,7Z,11E,13E,15Z,19Z-docosahexaenoic acid), resolvin D2 (7*S*,16*R*,17*S*-trihydroxy-4*Z*,8*E*,10*Z*,12*E*,14*E*,19*Z*-docosahexaenoic acid), and resolvin E1 (5S,12R,18R-trihydroxy-6Z,8E,10E,14Z,16E-eicosapentaenoic acid) (11). In addition to confirming the original structural assignments and potent anti-inflammatory and pro-resolving actions *in vivo* of resolvins, lipoxins, and maresins (12), recent findings demonstrate their potent actions in experimental colitis (13), arthritis (14), arthritic pain (15), ocular diseases (16), resolving adipose tissue inflammation (17), and diabetes (18). SPM share defining actions in resolving local inflammation; they each enhance macrophage uptake of cellular debris and apoptotic cells and limit further neutrophil recruitment to the site of injury and/or microbial invasion to bring about resolution (19, 20). The placement of these mediators within the resolution of inflammation as well as the state-of-the art definitions are reviewed earlier in Ref. (20, 21).

Pioneering studies conducted by Elie Metchnikoff paved the way to understanding the important role that macrophages play in orchestrating the host response. In his initial observations, Metchnikoff observed phagocytes surrounding and attempting to devour a splinter he had introduced into the transparent body of a starfish larva. Since then, the role of this process in mammalian systems, has been extensively studied where it is appreciated to be critical in both the maintenance of homeostasis and clearance of cellular debris, bacteria (6, 22), and apoptotic cells, a process termed efferocytosis (23). One of the defining actions displayed by pro-resolving mediators is the regulation of this fundamental process. Indeed, these mediators upregulate the ability of macrophages to phagocytose and kill bacteria, as well as to clear apoptotic cells and cellular debris (24). While pharmacologically these actions appear to be overlapping, recent studies demonstrate that at a biological level they are not. This is because the production of the different mediator families is regulated both temporally and in a tissue-specific manner (25–27). Furthermore, expression of the specific receptors, which form part of the G-protein-coupled receptor family, for each of the pro-resolving mediators is regulated in a cell type specific manner. For a detailed recent review of the biological actions and expression profiles of SPM receptors, the reader is directed to the following review (28). Recent evidence suggests that the clearance of apoptotic cells by macrophages leads to changes in the phenotype and functions of these cells (6, 7, 20, 29, 30). Thus, the aim of the present article is to discuss the role of pro-resolving mediators in regulating efferocytosis and the impact that this process plays in regulating macrophage lipid mediator (LM) profiles in order to shed light on the change in biological function (see Table 1 for a summary of the biological actions of SPM on macrophages).

**Table 1:** The role of SPM receptors in mediating the biological actions of these autacoids on macrophages.

Receptor	SPM	Biological action	Biological system	Reference
ALX/FPR2	RvD1	Suppression of AA-stimulated LTB_4_	Mouse bone marrow-derived Mϕ and zymosan-elicited peritoneal Mϕ	Fredman et al. (31)
	RvD1	Enhanced zymosan phagocytosis	Mouse bio-gel elicited Mϕ	Norling et al. (32)
	RvD1	Phagocytosis of zymosan and apoptotic PMNs	Human monocyte-derived Mϕ	Krishnamoorthy et al. (33)
	RvD1	Increased M2 polarization during I/R	Murine Kupffer cells	Kang and Lee (34)
	RvD1	Increases IL-10 levels	ALX/FPR2-overexpressing transgenic mice	Krishnamoorthy et al. (35)
			Human monocyte-derived Mϕ	
	RvD1	Reduces cigarette smoke extract promoted IL-6 and TNF-a	Human monocyte-derived Mϕ	Croasdell et al. (36)
	LXA_4_	increased transforming growth factor–β1	Murine Mϕ	Mitchell et al. (37)
	AT-LXA_4_	Increased efferocytosis		
	LXA_4_	Increase apoptotic PMN efferocytosis	Human monocyte-derived Mϕ	Godson et al. (38)
		Did not increase IL-8 and MCP-1		
		Attenuated PGE_2_-stimulated protein kinase A activation		
	LXA4	Reduced TNF-a production	Human monocyte-derived Mϕ	Pierdomenico et al. (39)
		Zymosan phagocytosis		

GPR32/DRV1	RvD1	Phagocytosis of zymosan	Human monocyte-derived Mϕ	Krishnamoorthy et al. (33)
		Efferocytosis of apoptotic PMNs		
	RvD1	Reduced IL-1β and IL-8 expression	Human monocyte-derived Mϕ	Schmid et al. (40)
		Reduced chemotaxis to chemerin, fMLF, and MCP-1		
	RvD1	Increase phagocytosis of bacteria	Human monocyte-derived Mϕ	Chiang et al. (41)
	RvD5			
	RvD1	Reduces IL-6 and TNF-a expression in elicited by cigarette smoke extract	Monocyte-derived Mϕ	Croasdell et al. (36)
	RvD3	Upregulate macrophage efferocytosis	Monocyte-derived Mϕ	Dalli et al. (25)
	AT-RvD3			

ChemR23/ERV1	RvE1	Increases IL-10 transcription and phagocytosis of microbial particles	Monocyte-derived Mϕ	Herova et al. (42)
	RvE1	Phagocytosis of zymosan A via AKT and ribosomal protein S6 phosphorylation	Monocyte-derived Mϕ	Ohira et al. (43)
	RvE1	Reduction of IL12p40 and TNF-a expression in cells incubated with LPS	Mouse peritoneal Mϕ	Ishida et al. (44)

GPR18/DRV2	RvD2	Enhanced phosphorylation of CREB, ERK1/2, and STAT3	Mouse exudate macrophages	Chiang et al. (45)
	RvD2	Enhanced phagocytosis of live *Escherichia coli* and apoptotic PMN	Human monocyte-derived macrophages	Chiang et al. (45)

### Microvesicles as Regulators of Efferocytosis and Macrophage LM Profiles

First thought to be byproducts of platelet activation carrying no significant biological activity, microvesicles are now increasingly appreciated to regulate critical aspects of the host immune response. Since their first description in platelets by Wolf in 1967 (46), the production of these microstructures has been described in many cell types including leukocytes, muscle cells and endothelial cells (47). They also carry distinct functions in both the initiation and resolution of acute inflammation [reviewed in Ref. (48)]. Microvesicles carry a wide range of molecular cargos including miRNA that are implicated in the regulation of hematopoiesis (49) as well as in protection during ischemia-reperfusion-mediated kidney injury (50). The pro-inflammatory cytokine IL1-β is also carried by microvesicles and its loading into these structures is thought to be one of the mechanisms by which this cytokine, which does not possess a secretion motif, is released from cells (51). Morphogens, such as Sonic Hedgehog, are also part of the cargo carried by subsets of these microvesicles, where microvesicles enriched in Sonic Hedgehog were found to promote angiogenesis and thus may play a role in tumor growth (52).

Recently, we found that neutrophil-derived microvesicles also display anti-inflammatory actions (53), carry precursors for the biosynthesis of pro-resolving mediators (29, 54), and display potent pro-resolving and host protective actions (54, 55). Since evolving self-limited inflammatory exudates produce functional microvesicles that also signal to stimulate resolution of inflammation in mice (54), we investigated whether these microvesicles also regulated macrophage efferocytosis. Indeed these microvesicles dose dependently regulated macrophage efferocytosis. Assessment LM-SPM concentrations in these microstructures demonstrated that neutrophil microvesicles, in addition to carrying precursors for the production of the pro-resolving mediators, also carried bioactive mediators including Protectin (PD) 1 (29).

Using a LM profiling approach, we found that microvesicles regulate macrophage LM-SPM profiles. Incubation of macrophages with neutrophil-derived microvesicles increased the biosynthesis of lipoxygenase- and cyclooxygenase-derived LM. These increased the biosynthesis of DHA-derived D-series resolvins, EPA-derived E-series resolvins, and the AA-derived lipoxins and prostanoids (29). Among the SPM that are upregulated, we observed significant increases in RvD5, MaR1, PD1, RvE2, and RvE1. Of interest, incubation of macrophages with G-protein inhibitors (pertussis toxin and cholera toxin) reduced SPM biosynthesis without altering prostanoid levels. Together, these results demonstrated that microparticles selectively stimulate macrophage SPM production in a G-protein-coupled receptor-dependent manner (29).

### Microvesicles Are a Nidus for Macrophage SPM Production during Efferocytosis

**Figure 1:**
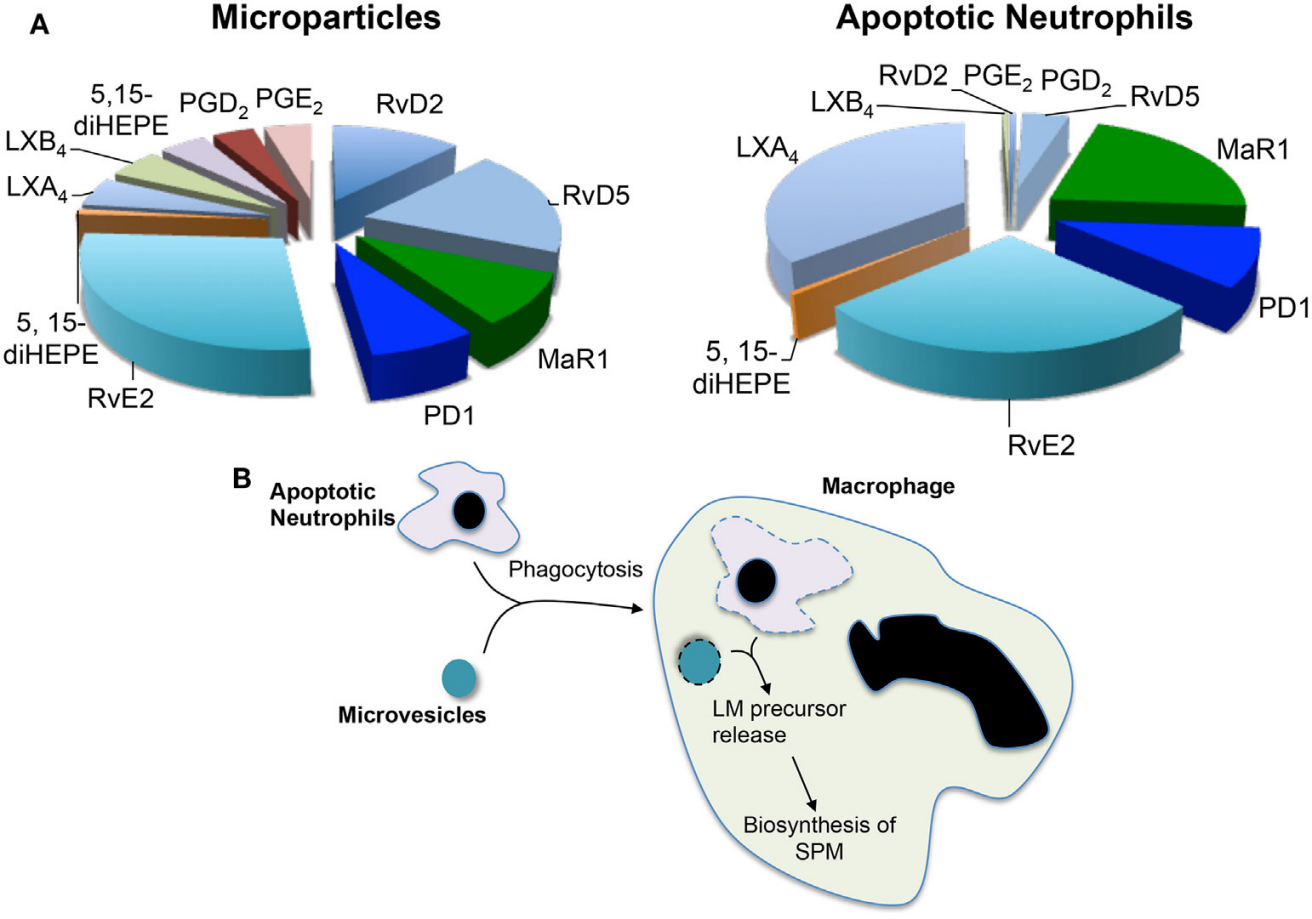
Microvesicles and apoptotic neutrophils are a nidus for specialized pro-resolving mediator (SPM) biosynthesis during efferocytosis. Microvesicles or neutrophils were enriched in deuterium-labeled essential fatty acids and the conversion of these essential fatty acids to lipid mediators and their pathway markers/precursors was assessed during efferocytosis using lipid mediator profiling. **(A)** Relative contribution to lipid mediator biosynthesis by microvesicles and apoptotic neutrophils. **(B)** Cartoon depicting the process of trancellular biosynthesis during efferocytosis.

Having observed increases in SPM levels and efferocyotosis when macrophages were incubated with neutrophil-derived microvesicles and that these microstructures carried elevated concentrations of several SPM precursors, the question arose whether microvesicles contributed to macrophage SPM biosynthesis by donating specific precursors. The potential contribution of microvesicles to transcellular biosynthesis was assessed during macrophage efferocytosis (29). For this purpose, we employed precursurs labeled with deuterium, which can be distinguished from endogenous precursors, to investigate the contribution of essential fatty acids derived from neutrophil-derived microvesicles. During efferocytosis, microvesicles were found to contribute to the production of *d*_5_-RvD2 and *d*_5_-RvD5 from the D-series resolvins and *d*_5_-PD1 from the protectin family (Figure 1). These results demonstrated that during macrophage efferocytosis transcellular biosynthesis contributes to LM production where both microvesicles contribute substrate utilized in the SPM production.

### Apoptotic PMN and Microvesicles Stimulate Macrophage SPM Production

Recent studies suggest that the process of efferocytosis reprograms macrophage responses altering cytokine production (7). Our studies demonstrate that the regulation of functional responses also extends to the production of both pro-resolving and pro-inflammatory LM profiles. Efferocytosis of apoptotic PMN by macrophages increases SPM biosynthesis, primarily, RvD1, RvD2, and LXB_4_. SPM biosynthesis during efferocytosis was further upregulated by microvesicles, increases that correlate with an enhancement in the uptake of apoptotic neutrophils by macrophages (29). Of note, addition of microvesicles to macrophages further upregulated macrophage biosynthesis of RvD2, LXB_4_, and RvE2, while reducing PGF_2α_ and TXB_2_ (29).

### Apoptotic PMN, a Nidus for Macrophage SPM Biosynthesis

Apoptotic cells were also found to supply precursors to macrophages for the biosynthesis of both eicosanoids and SPM. This contribution was determined using a similar approach to that used for microvesicles where apoptotic neutrophils were enriched in deuterium-labeled (*d*) precursors. These studies demonstrate that substrates obtained from apoptotic neutrophils were also utilized for the production of pro-resolving mediators, whereas *d*_5_-PD1 and *d*_5_-RvE2 were produced when the substrate was obtained from either microvesicles or apoptotic cells *d*_5_-MaR1 and *d*_8_-LXB_4_ were only identified in incubations with labeled apoptotic cells (29) (Figure 1). These results suggest that the origin of the substrate, and potentially its subcellular localization, may influence its contribution to specific mediator families.

### Apoptotic Cells Differentially Regulate LMs in M1 and M2 Macrophages

Because apoptotic neutrophils stimulate LM biosynthesis in macrophages, we investigated whether this finding held for different macrophage subtypes. Assessment of LM biosynthesis after apoptotic cell efferocytosis demonstrated that the uptake of apoptotic neutrophils by classically activated macrophages upregulated the SPM production and reduced prostanoid biosynthesis. Of note, incubation of apoptotic neutrophils with M2 macrophages reduced overall LM production, including SPMs (29). These results indicated that the regulation of SPM profiles by apoptotic cells is also cell type dependent and may reflect the distinct biological actions of diverse cell types within the initiation-resolution spectrum.

### Role the Role of G-Protein-Coupled Receptors in Mediating SPM Actions with Human Macrophages

SPM exert their biological actions via activating receptors of the G-protein-coupled receptor superfamily. RvD1, AT-RvD1, RvD3, AT-RvD3, LXA_4_, and AT-LXA_4_ activate the lipoxin receptor ALX/FPR2; in humans, these mediators also activate the orphan receptor DRV1/GPR32. RvD5 was also recently shown to activate DRV1/GPR32, while RvE1 binds to and activates ERV1/ChemR23; and DRV2/GPR18 mediates the biological actions of RvD2 (9, 23, 31, 39). Interested readers are referred to Ref. (9) for a detailed review on the biology of SPM receptors. In addition to displaying agonist actions to specific GPCR, RvE1, MaR1, and the MaR1 further metabolite 22-OH-MAR1 are also partial agonists/antagonists to LTB_4_ receptor BLT1.

Activation of the pro-resolving receptors by their cognate mediators occurs in a stereospecific manner with even minor changes to their structure resulting in a significant loss in their ability to bind and activate these receptors. Recent studies suggest that the expression of SPM receptors differs between macrophage subsets thus suggesting that pro-resolving mediators differentially regulate the biological actions of distinct macrophage subsets. Murine peritoneal macrophages express the murine homologs of the ALX/FPR2, DRV2/GPR18, and ERV1/ChemR23 that mediate the biologic actions of their cognate SPM in regulating pro-inflammatory cytokine production, upregulating bacterial clearance and efferocytosis of apoptotic cells (9). In humans, expression of ERV1/Cherm23 was recently suggested to be restricted to macrophages obtained under conditions leading to a classically activated phenotype (42).

### The Maresin Bioactive Metabolome in Human Macrophages

**Figure 2:**
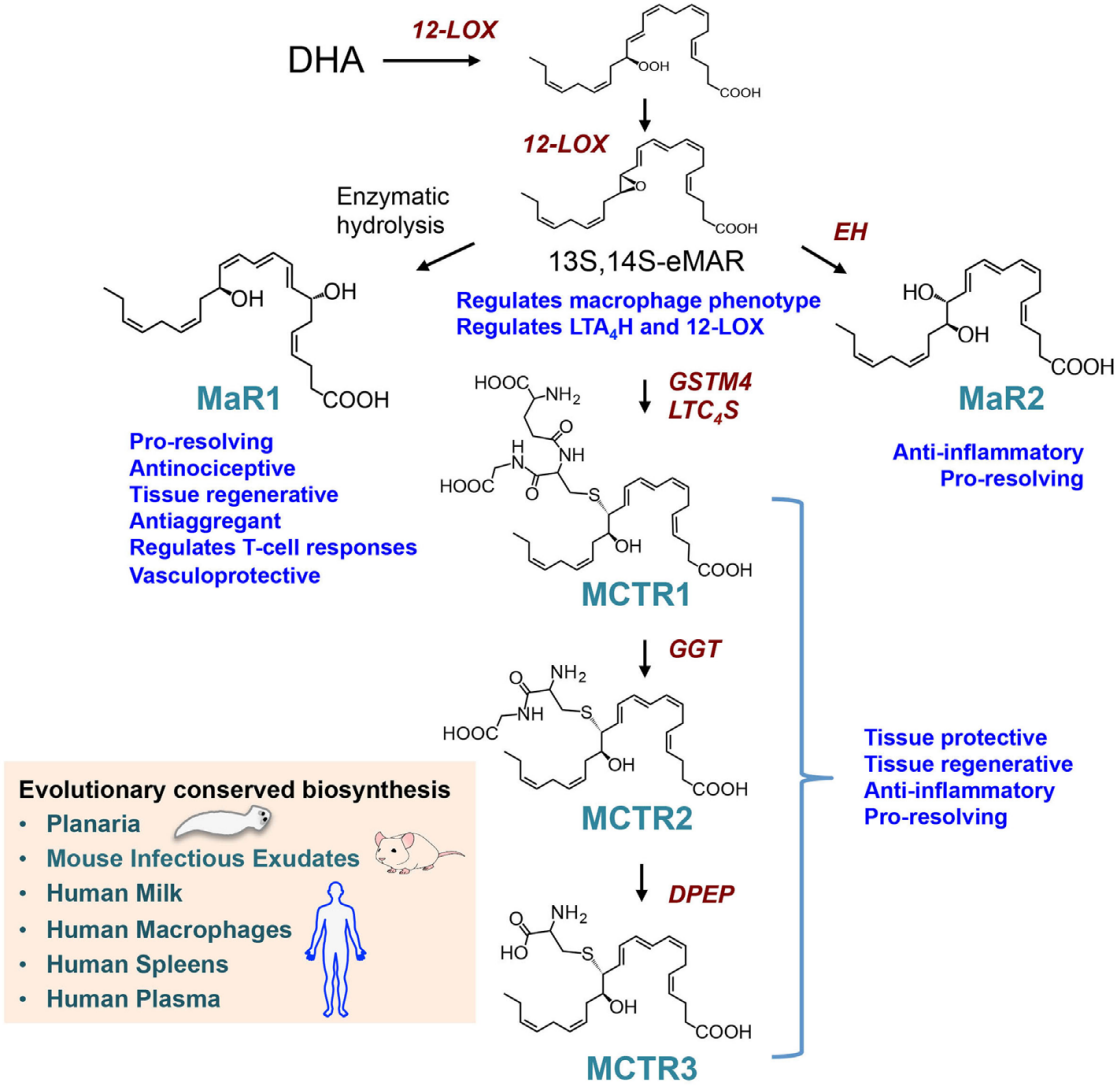
Biosynthesis and actions of the macrophage-derived Maresins. The pathway is initiated by 14-lipoxygenation of DHA to yield 14S-hydro(peroxy)-4Z,7Z,10Z,12E,14S,16Z,19Z-docosahexaenoic acid and then to 13S,14S-epoxy-4Z,7Z,9E,11E,13S,14S,16Z,19Z-docosahexaenoic acid (13S,14S-eMaR) reactions that are catalyzed by 12-LOX. This intermediate is then enzymatically hydrolyzed to 7R,14S-dihydroxy-4Z,7R,8E,10E,12Z,14S,16Z,19Z-docosahexaenoic acid (MaR1) or *via* an epoxide hydrolase (EH) to 13,14S-epoxy-4Z,7Z,9,11,13,14S,16Z,19Z-docosahexaenoic acid (MaR2). 13S,14S-eMAR is also substrate for Glutathione S-transferase MU 4 (GSTM4) and leukotriene C_4_ Synthase (LTC_4_S) yielding MCTR1 (13R-glutathionyl,14S-hydroxy-4Z,7Z,9E,11E,13R,14S,16Z,19Z-docosahexaenoic acid), which is then converted to MCTR2 (13R-cysteinylglycinyl,14S-hydroxy-4Z,7Z,9E,11E,13R,14S,16Z,19Z-docosahexaenoic acid) by gamma-glutamyl transferase (GGT) and to MCTR3 (13R-cysteinyl,14S-hydroxy-4Z,7Z,9E,11E,13R,14S,16Z,19Z-docosahexaenoic acid) by dipeptidase (DPEP).

Studies using LM metabolomics and self-resolving exudates uncovered a new family pro-resolving mediators produced by macrophages, these mediators were coined macrophage mediators in resolving inflammation (maresins) (56). In the biosynthesis of maresins, DHA is lipoxygenated at carbon 14 yielding 14S-hydro(peroxy)-docosa-4Z,7Z,10Z,12E,16Z,19Z-hexaenoic acid (14-HpDHA) that is further converted to an allylic epoxide, reactions carried out by macrophage 12-lipoxgenase (30, 57). Using stereocontrolled total organic synthesis, enantiomerically pure 13*S*,14*S*-epoxy-docosa-4*Z*,7*Z*,9*E*,11*E*,16*Z*,19*Z*-hexaenoic acid (13*S*,14*S*-eMaR) was prepared and its stereochemistry confirmed by nuclear magnetic resonance spectroscopy. In macrophages, this intermediate is enzymatically converted to the pro-resolving mediator MaR1 (Figure 2). The stereochemistry of MaR1 was recently established as 7*R*,14*S-*dihydroxydocosa-4Z,8*E*,10*E*,12*Z,16Z,19Z*-hexaenoic acid, using a matching approach with material obtained from biological systems and total organic synthesis (11). The biological actions of MaR1 were also confirmed using synthetic material that included regulating leukocyte responses including limiting neutrophil infiltration in murine peritonitis (ng/mouse range) as well as enhancing human macrophage uptake of apoptotic neutrophils (11).

The biological actions of MaR1 extend beyond the regulation of cellular trafficking, where studies using planaria demonstrate that this mediator accelerates tissue regeneration following surgical injury (11). Upon injury, planaria also produce MaR1 from deuterium-labeled (d_5_)-DHA in a lipoxygenase-dependent manner, since an inhibitor to this class of enzymes reduced MaR1 formation and also the ability of planaria to regenerate damaged tissues (11). Planaria are simple organisms capable of rapid regeneration, the process where in mammalian tissue macrophages play a central role. Factors involved in planaria tissue regeneration remained to be identified (58). Hence, we investigated whether MaR1 displays properties in controlling tissue regeneration. To this end, the anterior portions of planaria were surgically removed and the animals given MaR1. This accelerated tissue regeneration with the appearance of head regeneration was evident as early as 3 days post surgery. MaR1 addition at concentrations as low as 1 nM enhanced head regeneration as early as day 3 post injury (11).

MaR1 also possess potent anti-nociceptive actions, dose dependently inhibiting transient receptor potential cation channel subfamily V member 1 (TRPV1) currents in neurons, blocking capsaicin (CAP, 100 nM)-induced inward currents (IC_50_ = 0.49 ± 0.02) and reducing both inflammatory and chemotherapy-induced neuropathic pain in mice (11, 59, 60).

Of interest, 13*S*,14*S*-eMaR, the biosynthetic intermediate in the MaR1 metabolome, is also bioactive. Indeed this intermediate inhibits LTB_4_ formation by human leukotriene A_4_ hydrolase (LTA_4_H) ~40% (*p* < 0.05) to a similar extent as LTA_4_ (~50%, *p* < 0.05). Furthermore, LTA_4_H was not involved in converting 13*S*,14*S*-eMaR to MaR1 pointing to the involvement of a yet unidentified epoxide hydrolase in catalyzing this biosynthetic step. 13*S*,14*S*-eMaR also reduced (~60%; *p* < 0.05) arachidonic acid conversion by human 12-LOX and promotes macrophage phenotype switch toward an M2 profile with similar potency as MaR1 (30). Incubation of M1 macrophages with either 13S,14S-eMaR (10 nM) or MaR1 (10 nM) led to significant reductions in the M1 lineage markers CD54 and CD80 expression and a concomitant upregulation of the M2 lineage markers CD163 and CD206 (30). We also investigated the conversion of 13S,14S-eMaR to MaR1 by different human macrophage subtypes. 13S,14S-eMaR (2 µM) with M2 macrophages gave higher MaR1 levels then when the 13S,14S-eMaR was incubated with M1 macrophages. These results suggest that the MaR1 metabolome is central in regulating macrophage function and mediating the biological actions of these phagocytes (30).

### Identification of as Maresin Conjugates in Tissue Regeneration (MCTR) as Novel Regulators of Tissue Regeneration

Given the roles that macrophages play in the orchestrating wound healing, we questioned whether during the later stages of resolution these cells produce a distinct group of chemical signals that initiate tissue repair and regeneration. Using a systematic approach, we uncovered a group of peptide-lipid conjugated molecules that in addition to carrying the defining SPM pro-resolving actions, including the ability to regulate leukocyte trafficking and counter-regulate pro-inflammatory mediator production, also promote tissue repair and regeneration (8). The human macrophage 12-lipoxygenase is the initiating enzyme in the formation of these new signaling molecules, converting docosahexaenoic acid to 14S-HpDHA and then to 13S,14S-eMaR. Given that the initial biosynthetic steps are shared with MaR1, and carried a carbon 14 position alcohol, these bioactive molecules were coined as MCTR. The intermediate epoxide is converted to 13R-glutathionyl,14S-hydroxy-4Z,7Z,9E,11E,13R,14S,16Z,19Z-docosahexaenoic acid (MCTR1), a step that in human macrophages is catalyzed by Glutathione S-transferase MU 4 (GSTM4) and leukotriene C_4_ synthase (LTC_4_S) (61) (Figure 2). Of note, the two enzymes are shared with the cysteinyl LT pathway where they also catalyze the conversion of LTA_4_ to leukotriene C_4_ (LTC_4_). Each of these enzymes displayed different affinities to the two substrates, where LTC_4_S displayed a higher affinity to LTA_4_, whereas GSTM4 displayed a higher affinity toward 13S,14S-eMAR. These findings suggest that in addition to substrate availability, the relative expression of the two enzymes in one cell type may determine the balance between the inflammation-, contraction-, and stress-initiating LTC_4_ (7) in contrast with the tissue-regenerative pathway of MCTRs. MCTR1 is precursor to 13R-cysteinylglycinyl,14S-hydroxy-4Z,7Z,9E,11E,13R,14S,16Z,19Z-docosahexaenoic acid (MCTR2), a conversion catalyzed by gamma-glutamyl transferase (Figure 2). This mediator in addition to carrying pro-resolving and tissue regenerative actions is also precursor to the third member of the MCTR family of mediators, 13R-cysteinyl,14S-hydroxy-4Z,7Z,9E,11E,13R,14S,16Z,19Z-docosahexaenoic acid (MCTR3), a reaction that is catalyzed by dipeptidases (61) (Figure 2). Of note, these enzymes are also shared with the cysteinyl LT biosynthetic pathway suggesting that processes regulating substrate availability may be critical in determining the macrophage LM phenotype and therefore function.

The production and biological actions of members of the maresin family are evolutionary conserved with identification in planaria mouse infectious exudates, human breast milk, spleen, and plasma from sepsis patients (Figure 2). Using deuterium-labeled docosahexaenoic acid, we found that MaR1 was produced by planaria following surgical injury (11). MCTR1 and MCTR2 were also identified in regenerating planaria and incubation of these mediators with planaria accelerated tissue regeneration (8). This increase in the rate of regeneration was associated with an early upregulation of a number of genes that in these animals are associated with head-to-tail differentiation suggesting that these molecules form part of the tissue regeneration process engaged following injury in planaria. This is further supported by the finding that planaria express genes that are homologous to MCTR biosynthetic enzymes including GSTM4, which are upregulated in regenerating tissues (8). Inhibition of these enzymes using both genetic approaches and small molecule inhibitors reduced MCTR levels as well as the ability of planaria to regenerate. In mice, MCTRs were also found to regulate tissue repair and regeneration in lung tissue where administration of these mediators during ischemia-reperfusion-mediated injury protected the lung from leukocyte-mediated damage and upregulated the expression of molecules that are associated with cell proliferation and tissue repair in the lung (8).

Therefore, SPM emerge both as potent regulators of macrophage responses of interest during the resolution phase of acute inflammatory responses and effectors in macrophage-mediated responses. Since the means and methodologies to identify these mediators have only recently become widely available, a number of aspects of their biology in humans have only just started to be explored such as the production of these pro-resolving mediators in human tissues (62–64). In addition, a number of questions still remain to be addressed such as the when and where these mediators are produced in human tissues in health and disease and the relevance of resolution processes that may fail giving rise to human disease. In this context, we recently found that following either a systemic insult or local inflammatory stimulus LM biosynthesis is differentially regulated between males and females. In these studies, vaccination with typhoid vaccine lead to peripheral blood leukocyte activation and endothelia dysfunction in males but not in females. This was associated with an increase peripheral blood E-series resolvin and a downregulation in the levels of LTB_4_ in females (65). Local challenge using chantaridin led to a rapid resolution of the inflammatory response in females that was associated with an increased exudate RvD and decreased LTB_4_ concentrations. Differences in tissue SPM concentrations were also recently reported in patients with inflammatory arthritis where a correlation between synovial RvE2 concentrations and pain was observed, with higher concentrations of RvE2 in these inflammatory exudates correlating with lower pain in arthritic patients (66). Thus, these results underscore the role of SPM in controlling tissue inflammation in humans and the utility of measuring these mediators as a potential diagnostic tool in patient stratification.

Since resolution of inflammation is a fundamental process in all human tissues and that phagocytes and specifically macrophages play a central role in orchestrating this response as well as tissue repair and regeneration, immunoresolvents (such as resolvins, protectins, and maresins) may provide a novel therapeutic approach for diseases characterized by uncontrolled inflammation and failed resolution. In this context, recent findings demonstrate that enriching microvesicles with SPM may represent a novel therapeutic approach to control chronic inflammation and promote tissue regeneration. In minipigs, administration of a single dose of a novel pro-resolving nanomedicine, produced by enriching microvesicles in a lipoxin analog, markedly reduced periodontal disease, and promoted bone regeneration (67). These results, together with many other recent studies, emphasize that using agonists to reprogram the immune cells rather then inhibiting the inflammatory response may represent a novel paradigm to controlling inflammation without compromising the host immune response.

## Author Contributions

JD and CS contributed to manuscript preparation.

## Conflict of Interest Statement

The authors declare that the research was conducted in the absence of any commercial or financial relationships that could be construed as a potential conflict of interest.
